# Lung cancer and schwannoma - the pitfalls of positron emission tomography

**DOI:** 10.1590/S1806-37132014000300016

**Published:** 2014

**Authors:** Fernando Luiz Westphal, Luiz Carlos de Lima, José Correa Lima-Netto, Michel de Araújo Tavares, Felipe de Siqueira Moreira Gil

**Affiliations:** Federal University of Amazonas School of Medicine Getúlio Vargas University Hospital, Manaus, Brazil; Department of Thoracic Surgery, Federal University of Amazonas School of Medicine Getúlio Vargas University Hospital, Manaus, Brazil; Department of Thoracic Surgery, Federal University of Amazonas School of Medicine Getúlio Vargas University Hospital, Manaus, Brazil; Department of Clinical Medicine, Federal University of Amazonas School of Medicine, Manaus, Brazil; Federal University of Amazonas School of Medicine, Manaus, Brazil

To the Editor:

Imaging studies play an important role in the tumor-node-metastasis staging of lung cancer, particularly in the evaluation of tumor size/extent and regional lymph node involvement. There is a learning curve for interpreting positron emission tomography (PET) findings; PET allows the estimation of the metabolic activity of a lesion, therefore facilitating the differential diagnosis between benign and malignant disease, as well as the determination of the extent of malignancy. When used in the study of lung cancer, PET can give false-negative results in cases of decompensated hyperglycemia, small lesions, or lesions with low metabolic activity, among others.^(^ ^1^ ^,^ ^2^ ^)^ In addition, PET can give false-positive results in cases of inflammatory disease or concomitant tumors. 

**Figure 1:**
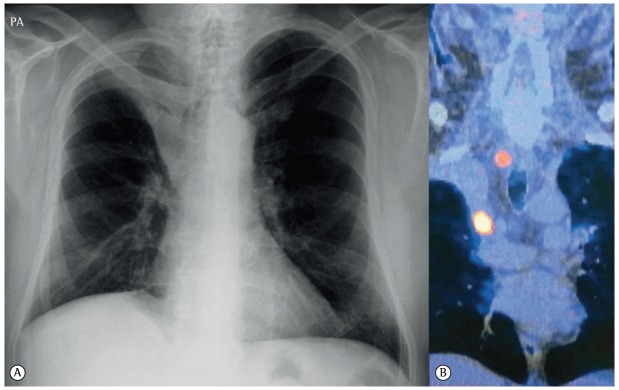
In A, posteroanterior chest X-ray showing a radiopaque triangular image in the right upper lobe determining cranial, fissural, and hilar retraction, suggestive of atelectasis. In B, coronal proton emission tomography and CT fusion images of two oval nodes with increased radiotracer uptake. One of the images represents a right upper lobe paramediastinal mass, which occluded the right upper lobe bronchus and therefore caused atelectasis. The other image represents a lesion in the right paratracheal lymph node station. Although the image was suggestive of lymph node enlargement, histopathological examination revealed a schwannoma.

We report the case of a 61-year-old male patient who, in October of 2011, presented to our emergency room with airway infection. A chest X-ray revealed right upper lobe atelectasis, which was confirmed by chest CT (Figure 1A).

Fiberoptic bronchoscopy showed an exophytic lesion in the right upper lobe orifice. The lesion caused total bronchial lumen occlusion and affected the secondary carina, without tracheal involvement. The histopathological findings were consistent with moderately differentiated squamous cell carcinoma. Blood and kidney function test results were normal, the exception being serum glucose levels, which were elevated. 

A PET scan showed a solid, lobulated paramediastinal lesion in the right upper lobe. The lesion measured 3.0 cm × 2.4 cm, and the standardized uptake value (SUV) was 12.2. The lesion occluded the right upper lobe bronchus and therefore caused the atelectasis. In addition, the PET scan showed a right-sided image that was suggestive of lymph node enlargement in the superior mediastinum and measured approximately 2.0 cm, the SUV being 3.8 (Figure 1B). 

**Figure 2:**
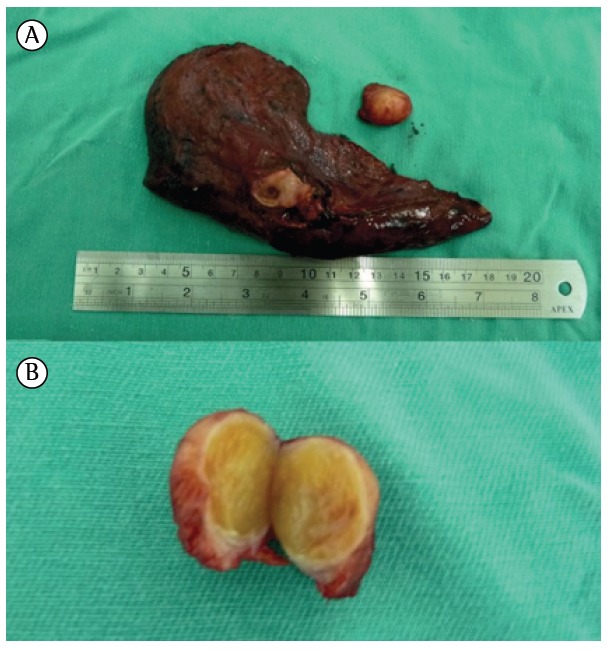
In A, photograph of the resected right upper lobe and mediastinal lesion. In B, photograph of the mediastinal lesion.

The patient underwent right upper sleeve lobectomy and radical lymphadenectomy, the affected lymph node being resected (Figure 2A). Histopathology of the lesion in the right lung apex revealed invasive, moderately differentiated squamous cell carcinoma measuring 3.2 cm × 1.6 cm, with angiolymphatic invasion, marked desmoplasia, necrotic foci, and scattered foci of squamous differentiation. Analysis of the nodule in the superior mediastinum revealed, instead of lymph node enlargement, a well-defined, benign encapsulated schwannoma (Figure 2B) without cellular atypia or necrotic areas. There were no postoperative complications, and the patient underwent chemotherapy. Screening tests performed one year later were negative for metastatic disease and recurrence.

Schwannomas (also known as neurilemmomas) and neurofibromas account for 95% of all benign neurogenic mediastinal tumors. A schwannoma is a mesenchymal neoplasm originating from Schwann cells of the nerve sheath. Although schwannomas commonly affect the mediastinum, they can be found in the abdomen, in the pelvis, and, much more rarely, in the chest wall. Schwannomas affect males and females alike, being benign in up to 90% of cases. In general, schwannomas are well defined and asymptomatic, being diagnosed incidentally, usually after the age of 30 years.^(^ ^3^ ^,^ ^4^ ^)^ Given that schwannomas show variable 18F-fluorodeoxyglucose (FDG) uptake, with the SUV ranging from 1.9 to 12.0, PET has limited utility in distinguishing between schwannomas and malignant peripheral nerve sheath tumors.^(^ ^5^ ^)^ In many cases, it is impossible to distinguish between a schwannoma and a malignant tumor before biopsy or surgery precisely because schwannomas can show high FDG uptake. 

Accurate staging is required for proper treatment of patients with lung cancer, staging being based on tumor size, regional lymph node involvement, and the presence of metastases. Although chest CT is considered the modality of choice for the diagnosis of intrathoracic metastases, there is no consensus regarding the study of metastases. PET-CT was introduced and developed as an integrated modality for accurate nodal staging and for detection of metastatic lesions in the entire body. Commonly, PET-CT is more effective than chest CT for lung cancer staging, assisting in detecting distant metastases that are not detected by traditional methods in 5-20% of patients; in addition, PET-CT can influence the treatment strategies and assist in predicting survival.^(^ ^6^ ^)^ Although the degree of regional lymph node involvement in our patient was classified as N_2_, surgery was recommended because only one lymph node was affected, and it was resectable. 

Given that schwannomas show highly variable FDG uptake-which is why it is difficult to differentiate between schwannomas and other tumors by means of images alone-PET findings in patients with lung masses should be interpreted carefully, as should lymph nodes with high SUV, which should be biopsied, in order to avoid an incorrect diagnosis or incorrect staging and the hazardous consequences of false-positive results.
