# Supplementary material for: Lung cancer and schwannoma - the pitfalls of positron emission tomography
Source: J Bras Pneumol. 2014 May-Jun;40(3):319–21. doi: 10.1590/S1806-37132014000300016 (PMC4109205; doi:10.1590/S1806-37132014000300016)
Supplement: Translation

# Translation: Câncer de pulmão e schwannoma - as armadilhas da tomografia por emissão de prótons

**Authors:** Fernando Luiz Westphal, Luiz Carlos de Lima, José Correa Lima-Netto, Michel de Araújo Tavares, Felipe de Siqueira Moreira Gil

**Affiliations:** Hospital Universitário Getúlio Vargas, Faculdade de Medicina, Universidade Federal do Amazonas, Manaus (AM) Brasil; Serviço de Cirurgia Torácica, Hospital Universitário Getúlio Vargas, Faculdade de Medicina, Universidade Federal do Amazonas, Manaus (AM) Brasil; Serviço de Cirurgia Torácica, Hospital Universitário Getúlio Vargas, Faculdade de Medicina, Universidade Federal do Amazonas, Manaus (AM) Brasil; Departamento de Clínica Médica, Universidade Federal do Amazonas, Manaus (AM) Brasil; Faculdade de Medicina, Universidade Federal do Amazonas, Manaus (AM) Brasil

Ao Editor:

Os exames de imagem costumam ter um papel importante no estadiamento pelo sistema tumor-nódulo-metástase das neoplasias pulmonares na avaliação dos fatores tumor e nódulo. A interpretação da *positron emission tomography* (PET, tomografia por emissão de pósitrons) passa por uma curva de aprendizado na avaliação dos achados; essa técnica permite estimar a atividade metabólica da lesão, facilitando assim o diagnóstico diferencial entre doenças malignas e benignas, bem como a extensão de uma doença maligna. A utilização da PET no estudo da neoplasia pulmonar pode induzir a resultados falso-negativos, tais como na presença de hiperglicemia descompensada, lesões pequenas ou lesões de baixa atividade metabólica.^(^ ^1^ ^,^ ^2^ ^)^ Ademais, podem ocorrer resultados falso-positivos, como nos casos de doenças inflamatórias ou na ocorrência de tumores concomitantes.

Relatamos o caso de um paciente do sexo masculino (61 anos) com infecção das vias aéreas que procurou atendimento em nossa unidade de urgência em outubro de 2011. A radiografia de tórax revelou atelectasia do lobo superior direito, confirmada pela TC de tórax. (Figura 1A)

**Figura 1:** Em A, radiografia do tórax na incidência posteroanterior demonstrando imagem triangular radiopaca no lobo superior direito que determina retração cranial, fissural e do hilo correspondente, sugerindo atelectasia. Em B, imagem de tomografia por emissão de prótons e TC com fusão de imagens no plano coronal demonstrando duas imagens ovaladas com hipercaptação do radiofármaco, sendo uma paramediastinal em lobo superior direito, que determinava oclusão do brônquio para o lobo superior direito e, consequentemente, atelectasia. A segunda imagem de lesão situava-se na cadeia paratraqueal direita sugerindo linfonodomegalia inicialmente mas confirmada pelo exame histopatológico como schwannoma.

À fibrobroncoscopia, foi evidenciada lesão vegetante em óstio do lobo superior direito, causando oclusão total da luz brônquica e envolvimento da carina secundária, sem acometimento de traqueia. O estudo histopatológico da lesão foi compatível com carcinoma de células escamosas moderadamente diferenciado. Com exceção da glicose sérica aumentada, os demais exames de função sanguínea e renal foram inalterados. 

A PET revelou uma lesão paramediastinal sólida em lobo superior direito, de contornos lobulados, medindo 3,0 × 2,4 cm e *standardized uptake value* (SUV, valor padronizado de captação) de 12,2. A lesão determinava oclusão do brônquio para o lobo superior direito, sendo assim responsável pela atelectasia. Ademais, o exame indicava a presença de uma imagem sugestiva de linfonodomegalia no mediastino superior, à direita, com cerca de 2,0 cm e SUV de 3,8 (Figura 1B).

O paciente foi submetido a uma lobectomia em manga do lobo pulmonar superior direito e linfadenectomia radical, com abordagem da cadeia comprometida (Figura 2A). A análise histopatológica da lesão em ápice pulmonar direito foi carcinoma de células escamosas moderadamente diferenciado, de caráter infiltrativo, medindo 3,2 × 1,6 cm, apresentando infiltração angiolinfática e constando acentuada desmoplasia e focos necróticos, além de esparsos focos de diferenciação escamosa. A análise do nódulo em mediastino superior identificou, em lugar de linfonodomegalia, um schwannoma benigno encapsulado (Figura 2B), bem delimitado, sem atipias celulares ou áreas de necrose. Não houve complicações pós-operatórias, e o paciente foi submetido à quimioterapia. Após um ano, os exames de rastreamento de metástase estavam negativos para doença metastática ou recidiva.

**Figura 2:** Em A, fotografia do lobo superior direito e da lesão mediastinal ressecados. Em B, fotografia da lesão mediastinal em destaque.

Dentre os tumores neurogênicos benignos que acometem o mediastino, os schwannomas (ou neurilemomas, como também são chamados) dividem com os neurofibromas a incidência de 95% dos casos. O schwannoma é uma neoplasia mesenquimal originada das células de Schwann da bainha dos nervos e comumente afeta o mediastino, podendo também ser encontrado em abdome, pelve e, com acentuada raridade, na parede torácica. Essas neoplasias costumam atingir igualmente homens e mulheres e são benignas em até 90% dos casos. Apresentam-se, em geral, bem delimitados e assintomáticos, sendo diagnosticados incidentalmente, geralmente após os 30 anos de idade.^(^ ^3^ ^,^ ^4^ ^)^ Como a absorção de 18F-fluordesoxiglicose (FDG) pelos schwannomas é variável, com SUV variando de 1,9 a 12,0, a PET possui uma limitação importante na distinção entre o schwannoma e as neoplasias malignas da bainha de nervos periféricos.^(^ ^5^ ^)^ É justamente devido a essa possibilidade de captação de altos valores de FDG que, muitas vezes, o schwannoma não pode ser distinguido de um tumor maligno antes da biópsia ou cirurgia. 

O tratamento adequado do câncer pulmonar necessita de estadiamento acurado, que é baseado no tamanho do tumor, no envolvimento linfonodal regional e na presença de metástases. Embora a TC de tórax seja considerada como a modalidade de escolha para o diagnóstico de metástases intratorácicas, não há consenso em relação ao estudo das metástases. A PET-TC foi introduzida e desenvolvida como uma modalidade integrada para o estadiamento nodal acurado e a detecção de lesões metastáticas de corpo inteiro. Comumente, a PET-TC tem uma eficácia maior que a TC de tórax para o estadiamento do câncer pulmonar, ajudando a identificar metástases à distância que não são encontradas por métodos tradicionais em 5-20% dos pacientes, e pode influenciar nas estratégias de tratamento e ajudar a predizer a sobrevida.^(^ ^6^ ^)^ Apesar de a doença neoplásica ser considerada neoplasia no estádio N_2_, a cirurgia foi indicada por tratar-se de um só gânglio comprometido, potencialmente ressecável.

Uma vez que a absorção de FDG pelos schwannomas é amplamente variável, dificultando sua diferenciação apenas por meio de imagens, os achados da PET no estudo de massas pulmonares devem ser analisados com cuidado, assim como os linfonodos com SUV alto que devem ser biopsiados, a fim de evitar um diagnóstico ou estadiamento errôneo e os efeitos deletérios de resultados falso-positivos.
